# Alcohol-Mediated Organ Damages: Heart and Brain

**DOI:** 10.3389/fphar.2018.00081

**Published:** 2018-02-13

**Authors:** Adam Obad, Ahmed Peeran, Janay I. Little, Georges E. Haddad, Sima T. Tarzami

**Affiliations:** Department of Physiology and Biophysics, Howard University, Washington, DC, United States

**Keywords:** alcohol abuse, cytokines, TNF-α, SDF-1, cardiovascular disease

## Abstract

Alcohol is one of the most commonly abused substances in the United States. Chronic consumption of ethanol has been responsible for numerous chronic diseases and conditions globally. The underlying mechanism of liver injury has been studied in depth, however, far fewer studies have examined other organs especially the heart and the central nervous system (CNS). The authors conducted a narrative review on the relationship of alcohol with heart disease and dementia. With that in mind, a complex relationship between inflammation and cardiovascular disease and dementia has been long proposed but inflammatory biomarkers have gained more attention lately. In this review we examine some of the consequences of the altered cytokine regulation that occurs in alcoholics in organs other than the liver. The article reviews the potential role of inflammatory markers such as TNF-α in predicting dementia and/or cardiovascular disease. It was found that TNF-α could promote and accelerate local inflammation and damage through autocrine/paracrine mechanisms. Unraveling the mechanisms linking chronic alcohol consumption with proinflammatory cytokine production and subsequent inflammatory signaling pathways activation in the heart and CNS, is essential to improve our understanding of the disease and hopefully facilitate the development of new remedies.

## Introduction

Alcohol is one of the most commonly abused substances in the United States. The effect of alcohol on organ systems of the body extends beyond the liver, where it is metabolized, to include the central nervous system, cardiovascular system, kidneys, lung, gastrointestinal tract, pancreas, and the immune system (Rodriguez et al., 2004; Walker et al., 2013). Due to its ability to distribute throughout most fluid compartments of the body (Dubowski, 1985), the chronic consumption of ethanol leads to cell injury in nearly every tissue, specifically cardiac tissue. Unfortunately, the incidence of heart disease due to chronic alcohol consumption continues to rise owing to the increased chronic alcohol drinking habits among American youth. In fact, individuals who abuse alcohol have been shown to have a high percentage of cardiovascular disease; the leading cause of death, disability, and healthcare expense in the United States.

**Figure 1:**
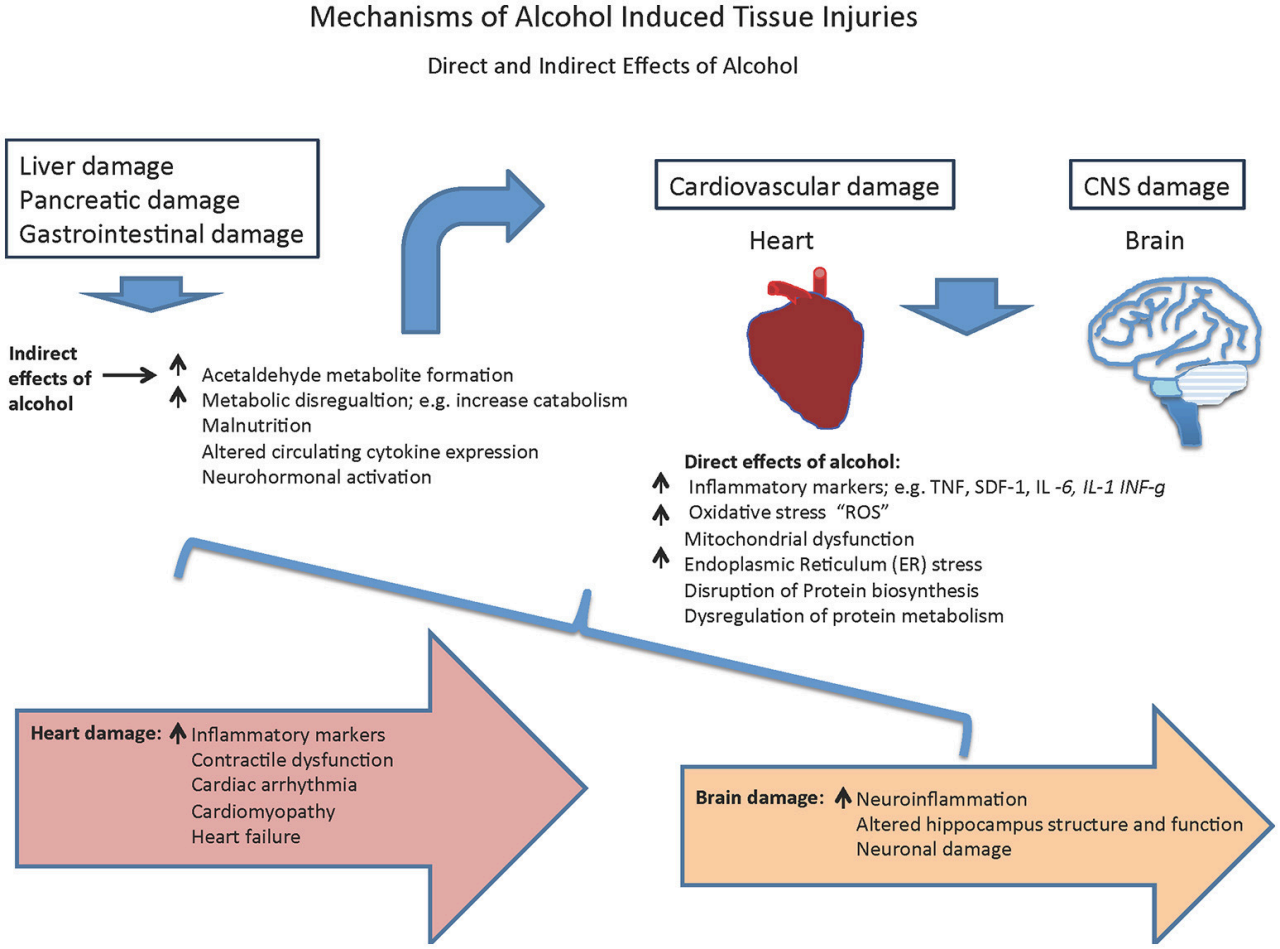
Mechanisms of alcohol-induced tissue injuries.

Conventionally, liver injury due to alcohol consumption has been studied in depth. The liver is the main metabolizer of alcohol and hence alcohol has harmful effects on liver cells. The Long periods of alcohol abuse can cause the liver to become inflamed, swollen or scarred (cirrhosis; Louvet and Mathurin, 2015). However, recently there has been increasing momentum in studying alcohol-induced cardiac and CNS injury. Particularly, the relationship between inflammation and inflammatory markers with alcoholic heart disease has gained much attention lately. This review summarizes the recent progress on the deleterious effects of alcohol abuse on a person's general health with special focus on the heart and brain (Figure 1). These organs consist of permanent cells, where the mechanisms underlying ethanol mediated damage is still not clear. Understanding the nature of the deleterious effects of alcohol on these organs may provide new insights on how to manage and/or combat effectors of alcohol-induced cell injury.

## Deleterious effect of chronic excessive alcohol consumption

Generally speaking, mechanisms of alcohol-related organ injury were attributed to increases in oxidative stress, impairments in methylation, unusual posttranslational modifications of proteins, dysregulation in lipid metabolism and signal transduction pathways, all of which ultimately affect cell survival and function (Song et al., 2014). However, this review highlights the contribution of inflammatory processes in alcohol-mediated tissue damage specifically in the context of cardiac and CNS damage whose injury mechanisms are not yet fully understood. Postmortem studies of both human alcoholic brains and basic animal models have demonstrated that heavy alcohol use is associated with an increased incidence of myopathy and neuropathy suggesting heart and brain changes (Crews et al., 2004; Liu et al., 2004, 2006; Remick, 2013; Fernandez-Sola and Planavila Porta, 2016). Interestingly heavy alcoholism is also associated with increased cytokine secretion and inflammation in these organs, reinforcing the possibility that cytokines play a pivotal role in alcohol-related brain and heart damages (Tables 1–2).

**Table 1:** Neuroinflammation and associated cytokines.

**Cytokine**	**Effect(s)**	**Mechanism(s)**	**Study(s)**
TNF-α	Brain: inflammation	Promotes inflammation, ultimately leading to neuronal cell death	Janelsins et al., 2008
TNF-α	Brain: immune modulation	Administration of TNF-α inhibitor, etanercept, improved the AD patient Mini-Mental State Examination (MMSE) outcomes	Tobinick, 2006; Tobinick et al., 2006
TNF-α	Brain injury: neuronal degeneration	TNF-α potentiate glutamate-mediated cytotoxicity in astrocyte leading to neuronal degeneration	Pickering et al., 2005
TNF-α	Many systems including brain	Controversial role as to whether it activates or limits inflammation	Akiyama et al., 2000a,b
TNF-α	CNS: neuromodulator	TNF is synthesized by neurons and act as a neuromodulator	Breder et al., 1993
NFkB & TNF-α	Many systems including brain	Ethanol increases DNA binding to (NFkB)	Zou and Crews, 2006
NFkB & TNF-α	Brain: inflammation	High levels of NFkB in alcoholic brains in postmortum humans	Liu et al., 2004, 2006; Okvist et al., 2007; Qin et al., 2008
NFkB & TNF-α	Brain: inflammation	Increased NFkB leads to increased TNF-α, leading to inflammation	Okvist et al., 2007; Zou and Crews, 2010
NF-κB, TNF-α, & SDF-1,	Brain: cell death in primary astrocytes	TNF-α cytotoxicity leads to activation of NF-κB via SDF-1 activation, which leads to increases the production of other cytokines and chemokines and triggers cell death in primary astrocytes	Han et al., 2001
TNF-α & SDF-1	CNS: immune cells modulation	TNF-α induction of SDF-1 limits immune cells from entering the CNS	Blazevski et al., 2015
SDF-1	Brain: neuronal cell death	SDF-1 and its receptor might contribute to neuronal apoptosis in the AIDS dementia complex	Rostasy et al., 2003

**Table 2:** Potential mechanism(s) underlying alcohol-induced heart damages.

**Factor**	**Effect(s)**	**Mechanism(s)**	**Study**
Alcohol	Reduction in contractility and ejection fraction, and myopathies including hypertrophy and myofibrillal abnormalities	Multifactorial	Preedy et al., 2003; Kannan et al., 2004; Li and Ren, 2008; Umoh et al., 2014
Alcohol	Stress on the heart	Circulatory changes	Ji, 2012
Alcohol	Hypertrophy and increase the assembly of sarcomeres	Increased circulatory growth factors, cytokines, and neurohormonal activation	MacHackova et al., 2006; Ji, 2012
Alcohol	Alcoholic cardiomyopathy	Decreases the activation of survival pathways Akt	Zhang et al., 2003; Walker et al., 2013; Umoh et al., 2014
Alcohol	Alcoholic cardiomyopathy; agonist and time dependent, activation with acute and deactivation after chronic exposure	Decrease the activation of the anti-apoptotic pathway related to the Mitogen Activated Protein kinases (MAPK)	Aroor et al., 2002; Aroor and Shukla, 2004; Li et al., 2006
Alcohol	Alcoholic cardiomyopathy and heart failure	Cardiomyocyte apoptosis	Fernandez-Sola et al., 2006
Heavy alcohol consumption	Ventricular arrhythmias and sudden cardiac death (SCD)	Multifactorial	Wannamethee and Shaper, 1992; Leaf et al., 2003
5-10 years of heavy drinking	Alcohol-related myocytes disease was associated with left ventricle changes such as dilation, increased mass, and normal or reduced thickness of the wall	Multifactorial	Adams and Hirst, 1990; Chen et al., 2000; Jankala et al., 2001; Vary and Deiter, 2005
Heavy drinking	Reduced ejection fraction and decreased contractility; this effect is correlated with duration and quantity of consumption	Progressive disarrangement and fragmentation of the myocytes' sarcomeres, defective pumping machinery of the heart	Danziger et al., 1991; Vary et al., 2001
In alcoholic population	Heart failure; reduction in stroke volume and low ejection fraction	Multifactorial	Patel et al., 1997a; Urbano-Marquez and Fernandez-Sola, 2004, 2005
Heavy alcohol consumption	Alcohol-induced heart disease	Left ventricle dysfunction	Haddad et al., 2008; Walker et al., 2013; Umoh et al., 2014

It is reported that alcohol abuse is associated with an increased incidence of a number of infectious diseases (Moss et al., 2003; Romeo et al., 2007). The effects of alcohol on the immune system involve various types of immune cells and their interactions. Using a murine model that parallels human alcoholism, Latif et al., has shown that alcohol impairs Th1-mediated delayed hypersensitivity (DTH) reactions (Wang et al., 2001; Latif et al., 2002). Furthermore, alcohol consumption in mice suppresses the cytolytic activity of natural killer (NK). Using mouse models, wherein mice were exposed to ethanol for relatively short periods of time (1–2 weeks), a significant reduction of splenic NK cells number was observed; this reduction directly correlated with a reduced NK cell activity *in vitro* (Meadows et al., 1992; Spitzer and Meadows, 1999). Spitzer and Meadows have shown that alcohol impairs the specific target-induced release, activity, and expression of cytolytic factors e.g., granular proteases and perforin, in fresh NK and IL2-stimulated NK in response to tumor targets (Meadows et al., 1992; Spitzer and Meadows, 1999). This ties into another aspect of alcoholic damage in which high doses of alcohol consumption can directly suppress a wide range of immune responses, causing increased susceptibility to certain diseases e.g., cancer (Lundberg and Passik, 1997).

Alcoholism's effects on the brain and heart vary and are influenced by a wide range of variables including the amount of alcohol consumed, the age at which the person began drinking, the duration of drinking, and several other factors (Hommer, 2003; Zeigler et al., 2005; Haddad et al., 2008; Lemstra et al., 2008). Thus, this review is mainly devoted to analyzing the underlying mechanisms of damages associated with heavy alcoholism focusing on brain and heart.

## Chronic excessive alcohol consumption and neurologic disorders

It is well-documented that heavy alcohol consumption provokes an array of degenerative pathologies, but the effectors that couple alcohol exposure to regulated forms of cell death are poorly understood. Several factors determine the degree of alcohol effect on the brain including: the frequency at which a person drinks, the amount of consumption, the duration of drinking, family history of alcohol consumption, the prenatal alcohol exposure, genetic background, and the person's general health status (Estruch et al., 1993; Keenan et al., 1997; Cervilla et al., 2000; Baumgartner et al., 2002; Oscar-Berman and Bowirrat, 2005). Not only does excessive alcohol consumption causes a person to be intoxicated, but it also increases his/her risk of experiencing blackouts. Blackouts are “intervals of time for which the intoxicated person cannot recall key details of events, or even entire events” (Hartzler and Fromme, 2003; Kahkonen et al., 2003).

Brain shrinkage is a common sign of brain damage; studies have shown that alcoholic men and women have significant shrinkage of their brains compared to non-alcoholic subjects. Similarly alcohol consumption causes impairment in their memory as well as their learning abilities (Wilkinson and Carlen, 1980; Eckardt et al., 1998; Nolen-Hoeksema and Hilt, 2006). Interestingly, in these studies women have shown higher susceptibility to these health problems, therefore they are more vulnerable. Unfortunately, these studies comparing men and women have been inconclusive.

Besides the overall effect on the brain, alcoholism can be detrimental to the cerebellum. Harm to the cerebellum mainly leads to loss of muscular coordination. The staggering gait and associated imbalance is a manifestation of the muscular coordination loss due to cerebellar damage (Oscar-Berman and Pulaski, 1997). The effect of alcohol could extend to the peripheral nervous system leading to peripheral neuropathy reflected as numbness and weakness in the hands and feet.

Korsakoff's syndrome (KS) is another recognizable complication of chronic excessive alcohol consumption (Oscar-Berman and Pulaski, 1997). In this syndrome, patient has memory loss with difficulty remembering their daily activities shortly after their occurrence, for which they are stuck in their old memories. The clinical manifestation of this syndrome is believed to be influenced by the genetic makeup of alcoholics, who might be predisposed to developing several forms of amnesia. Furthermore, some of these patients cannot properly process thiamine or vitamin B efficiently due to an enzyme deficiency (Oscar-Berman and Pulaski, 1997). The high alcohol consumption and low utility of thiamine can lead to brain damage, which causes the amnesia. Although these alcoholic patients experience amnesia, this does not directly affect their long-term memory, which includes their intelligence, as well as memories formed before the development of the disease (Oscar-Berman and Pulaski, 1997; Nixon, 2006).

There is strong evidence supporting the fact that alcohol exposure during developmental stages results in devastating selective neuronal damage resulting in profound central nervous system (CNS) deficits. The severity of this damage depends on the duration, and frequency of exposure to ethanol during gestation. There is strong evidence that during prenatal development alcohol exposure has negative consequences, however, the causes ethanol-induced neurodegeneration are poorly understood. Alcohol has been linked to hyper-inflammation, reactive oxygen species (ROS) generation and ultimately neuronal death (Ke et al., 2011; Fernandez-Lizarbe et al., 2013). Recent evidence appears to support an involvement of ER stress in alcohol-induced neuron toxicity. Increase of ER stress response proteins, i.e., ATF6, CHOP, GRP78, and mesencephalic astrocyte-derived neurotrophic factor as well as the phosphorylation of IRE1, eIF2α, PERK, and PKR were also detected within 24 h after the ethanol exposure (Bommiasamy et al., 2009; Ke et al., 2011). It has also been suggested that alcohol triggers an inflammatory response and causes a hyper-inflammation status, which mediates its damaging effects. Fernandez-Lizarbe et al. (2013), demonstrated that in microglial cells ethanol up-regulates TLR4 and TLR2 recruitment into lipid rafts-caveolae, mimicking their activation by their ligands (Fernandez-Lizarbe et al., 2013). This in turn will trigger induction of inflammatory mediators, generation of ROS and ultimately leads to neuroinflammation and neuronal cell death.

Chronic alcohol abuse also affects the integrity of blood-brain barrier (BBB), which can increase the influx of proinflammatory mediators and leukocytes into the brain. The BBB consists of the mainly of specialized endothelial cells lining the cerebral blood vessels, surrounded by pericytes, astrocytical processes, and neurons. It is known that alcohol can directly pass through the BBB, ultimately reaching the brain cells and causing subsequent neuronal toxicity, however the underlying mechanism is not fully understood. It was indeed suggested that chronic ethanol exposure induces oxidative stress and neuroinflammation in part by affecting platelet endothelial cell adhesion molecule-1 (PECAM-1) expression.

Endothelial PECAM-1 is a cell adhesion molecule that allows for the interaction of immune cells and the endothelium which facilitates the transmigration of leukocytes and thus contributes to the endothelial cell permeability barrier (Lertkiatmongkol et al., 2016); endothelial PECAM-1 (not Leukocytic PECAM-1) is the key regulatory component in this process (Wong and Dorovini-Zis, 1996; Lertkiatmongkol et al., 2016; Mandyam et al., 2017). It was reported that PECAM-1 may contribute to BBB damage, decline in oligodendrogenesis, demyelination and subsequent cognitive dysfunction (Mandyam et al., 2017). More over PECAM-1 could enhance the expression of NF-kB, a proinflammatory transcription factor which is produced under conditions of oxidative stress. Upregulation of NF-kB signaling further enhances the immune response, and this, in conjunction with impaired vascular endothelial integrity, could further facilitate the infiltration of leukocytes into the brain (Mandyam et al., 2017).

A review of the literature demonstrates that PECAM plays multiple pro-inflammatory roles in the current clinical context; however, there is evidence to suggest that there are also anti-inflammatory mechanisms regulated by endothelial PECAM-1. PECAM-1 was found to increase the resilience of the endothelial cell barrier and to suppress the proinflammatory cytokines which in turn dampened leukocyte infiltration into the brain (Privratsky et al., 2010). Although, the mechanisms through which PECAM-1 regulates these opposing functions is not fully understood, it is clear that PECAM-1 plays an important role in maintaining BBB integrity and may contribute to pathological inflammatory phenotype seen in alcoholism.

## Neuroinflammation and cytokine levels

The importance of pro-inflammatory cytokines has been implicated in neuroinflammation, neuroprotection, and neurogenesis (Choi et al., 2008). Of all cytokines the most widely investigated pro-inflammatory cytokines are Interleukin-1β (IL-1β), interleukin-6 (IL-6), Interferon-gamma (IF-γ) and tumor necrosis factor-α (TNF-α; Munoz-Fernandez and Fresno, 1998; Bauer et al., 2009). Unlike physiological conditions where cytokines' levels are kept at low levels, pathological conditions can increase their levels up to 100-folds the normal levels (Pitossi et al., 1997; Lee et al., 2002). For example, during trauma or injury to the nervous system, glial cells are shown to be activated, which leads to the production of a plethora of cytokines (Munoz-Fernandez and Fresno, 1998; Sheng et al., 2011). An important role of cytokines pertaining to their pro-inflammatory effect is their ability to upregulate cell adhesion molecules leading to alteration of the BBB integrity.

Among the inflammatory mediators, special interest has been paid to TNF-α. In addition to its key proinflammatory role, this pleiotropic cytokine mediates several pathological and physiological processes such as inflammation, differentiation, and apoptosis (Table 1; Janelsins et al., 2008). Referred to as the “master regulator,” TNF-α initiates and modulates the immune response in many systems including the brain (Tobinick, 2006; Tobinick et al., 2006). TNF-α role in normal brain functions has not been fully understood. Yet, many postulated hypotheses about many important CNS-related functions; an example of which, is its role in the development of hippocampal neurons (McCoy and Tansey, 2008). TNF-α levels elevation was implicated in ischemic and traumatic brain injuries (Pickering et al., 2005). Furthermore, TNF-α has an essential role in starting and regulating the steps of cascades of several cytokines during inflammation (Tobinick et al., 2006; Blazevski et al., 2015). However, the exact physiological role of TNF-α posed a challenge to scientists due to its low levels in healthy brains. The source of TNF-α and other inflammatory cytokines and neurotoxic substances during inflammation have been suggested to be by activated microglial cells (Breder et al., 1993). During the neuroinflammatory status in many neurological disorders, microglial cells release a plethora of newly synthesized TNF-α. However, the role of TNF-α remains controversial as to whether it exacerbates the neuronal injury or actively limits injury (Akiyama et al., 2000a,b). Hence, some clinical and preclinical studies postulated that interfering with TNF-α action in the brain might reduce the severity and delay the progression of many neurodegenerative disorders (McCoy and Tansey, 2008).

In Alzheimer's disease (AD), not only is there abnormal production of TNF-α, but TNF-α has also been linked to the pathological neuromodulation of the disease (McAlpine et al., 2009; Swardfager et al., 2010). Significantly elevated levels of TNF-α were associated with early onset AD; with 65 years of age as the cut of point between early and late onset AD (Gezen-Ak et al., 2013). When serum levels of TNF-α were compared between patients who have severe or mild forms of AD, higher levels of TNF-α seems to correlate with increased severity and faster rate of the disease progression (McCoy and Tansey, 2008).

Alcohol is well-known for its dysregulation of cytokines levels in several body organs such as the liver, brain, lung, and plasma. It has been postulated that such changes are responsible for undesirables CNS alterations, which results in long term effects in behavior and permanent neurodegenerative effect. Hence, understanding the role of such cytokines is essential to determine the exact pathogenesis of alcohol-associated neurological disorders (Achur et al., 2010). The aforementioned dysregulation of cytokines production was also found to be abnormal during monocytes response to several pathogens as well. The exact mechanism of alcoholic disruption of cytokines production and inflammation is not fully understood, yet, many pathways were suggested to contribute to the pathogenesis of alcoholic diseases (Manzo-Avalos and Saavedra-Molina, 2010; Crews et al., 2015).

Along with other cytokines, TNF-α was found to be dysregulated by alcohol-related tissue destruction as is evident by the abnormal levels of circulating cytokines in alcoholic patients (Achur et al., 2010). It was shown that ethanol treatment increases DNA binding to NFkB transcription factor (Zou and Crews, 2006). NFkB activation is proinflammatory and leads to increases in the transcription of TNF-α production, it has also shown to be associated with ethanol-induced inflammation (Okvist et al., 2007; Zou and Crews, 2010). Furthermore, postmortem studies of human alcoholic brain shows higher expression of NFkB transcription and related proinflammatory genes in the brains of alcoholics suggesting a likely link between the neuroinflammation and alcohol-induced neurodegeneration (Liu et al., 2004, 2006), similar results were also found in animal studies which have associated alcohol intake to proinflammatory gene expression and brain neuronal cell death (Okvist et al., 2007; Qin et al., 2008).

## Chronic excessive alcohol consumption and heart damages

Alcohol can be beneficial or harmful and may affect the heart and whole cardiovascular system in many ways. Some of the cardiovascular diseases that are associated with heavy drinking are: cardiomyopathy, cardiac arrhythmias, hypertension, atherosclerosis and heart failure (Table 3; Hansson et al., 2002; Steinbigler et al., 2003; Guo et al., 2012; Da Silva et al., 2013). In this section we are mainly focusing on cardiac abnormalities that are associated with heavy alcohol consumption. There is sufficient documentation supporting the fact that excessive chronic consumption of alcohol increases the risk to develop several cardiovascular pathologies such as: reduction in contractility and ejection fraction, and myopathies including hypertrophy and myofibrillal abnormalities (Table 2; Preedy et al., 2003; Kannan et al., 2004; Li and Ren, 2008; Umoh et al., 2014). Alcohol may change the circulatory hemodynamics resulting in stress on the heart (Ji, 2012). The stressed myocytes undergo hypertrophy and increases the assembly of sarcomeres in response to the high demand of cardiac output; such effect is likely caused by increased circulating growth factors and cytokine along with neurohormonal activation (Table 2; MacHackova et al., 2006; Ji, 2012). Furthermore, alcohol-induced heart disease is believed to result from the left ventricle dysfunction that results from heavy chronic alcohol drinking habits (Table 2; Haddad et al., 2008). Many of these cardiovascular diseases associated with alcohol abuse can eventually develop to heart failure and unfortunately; this becomes a major public health issue and a burden on the health system (Djousse and Gaziano, 2008a,b; Haddad et al., 2008).

**Table 3:** Potential mechanisms underlying alcohol consumption and cardiovascular disease.

**Cardiovascular disease**	**Potential mechanism(s)**	**Studies**
Hypertension	Increase in catecholamine secretion	Da Silva et al., 2013
Atrial fibrillation	Increase in reactive oxidative metabolites.	Steinbigler et al., 2003
Atherosclerosis	Activation of innate and adaptive immunity, Presence of inflammatory mediators e.g., TNF and IFN-gamma	Hansson et al., 2002
Alcoholic cardiomyopathy	Increase in pro-inflammatory effect, neurohormonal activation, Metabolic changes, acetaldehyde accumulation, impaired protein synthesis	MacHackova et al., 2006; Guo et al., 2012; Ji, 2012
Heart failure	Cardiovascular disease, coronary artery disease, malnutrition, vitamin deficiency	Faris et al., 2003; Urbano-Marquez and Fernandez-Sola, 2004, 2005; Fernandez-Sola et al., 2006

According to Djousse and Gaziano, approximately half a million Americans are diagnosed with heart failure each year and some of those cases are associated with alcohol consumption (Djousse and Gaziano, 2008b). Additionally, the effect of chronic alcohol intake on cardiomyocytes was correlated with the dose of consumption (Table 2; Haddad et al., 2008; Umoh et al., 2014). As opposed to heavy drinking, which was proven to increase the risk to develop cardiac failure, chronic low or moderate consumption was associated with lower risk of developing heart failure (Kagan et al., 1989; Wannamethee and Shaper, 1992; Wannamethee et al., 1995; Albert et al., 1999). The defining cut point between heavy and light-to-moderate consumption is different between men and women; being 1 cup per day for the latter and 2 cups per day for the earlier (Djousse and Gaziano, 2008b). Unfortunately, not many studies were dedicated to reveal the exact risk of developing heart failure in relation to specific types of alcoholic beverages (Djousse et al., 2004a). A link between the incidence of ventricular arrhythmias and sudden cardiac death (SCD) and heavy drinking has been suggested (Wannamethee and Shaper, 1992; Leaf et al., 2003). In patients with history of excessive drinking for 5–10 years, alcohol-related myocytes disease was associated with left ventricle changes such as dilation, increased mass, and normal or reduced thickness of the wall (Adams and Hirst, 1990; Chen et al., 2000; Jankala et al., 2001; Vary and Deiter, 2005). However, current literature does not have sufficient data to determine the duration and quantity required to manifest the frank picture of alcoholic cardiomyopathy. However, many retrospective studies found a minimum history of 10 years of excessive consumption among symptomatic alcoholic heart failure patients. Abstinence from drinking in such patients may reduce further risk and improve survival (Nicolas et al., 2002).

Cardiac disease remains an essential burden that causes death in chronic alcoholic population (Kannel, 1998; Banks, 2008). Excessive and prolonged alcohol drinking can lead to cardiovascular injuries known as alcoholic heart muscle disease leading to reduced ejection fraction and decreased contractility; a syndrome that is not usually caused by short-term consumption (Danziger et al., 1991; Vary et al., 2001). Correlating with the quantity and duration of drinking, the progressive disarrangement, functional loss, and fragmentation of the myocytes' sarcomeres seems to interfere with pumping machinery of the heart and reduces its performance (Vary et al., 2001). However, causal relationship between heaving drinking and alcoholic heart disease remains equivocal and debatable (Richardson et al., 1986). Unlike with liver disease, no studies have determined the relation between the quantity of consumption and alcoholic cardiomyopathy, rather it is a clinical diagnosis in heavy drinkers who have no other obvious causes for their heart disease (Richardson et al., 1986).

Despite the lack of clear understanding of the exact mechanism by which chronic alcohol consumption causes heart failure, stroke volume reduction and low ejections fraction was found in alcoholic population (Patel et al., 1997a,b; Urbano-Marquez and Fernandez-Sola, 2004, 2005). Such effect was found to be related to the dose of alcohol, regardless of the presence or absence of coronary artery disease, malnutrition, and/or vitamin deficiencies (Faris et al., 2003; Urbano-Marquez and Fernandez-Sola, 2004; Fernandez-Sola et al., 2006). Hence, alcoholic cardiomyopathy occurs in chronic alcohol heavy drinkers, who do not manifest any other cause for their underlying pathology (Wodak and Richardson, 1982; Richardson et al., 1986).

## Alcohol and cardiomyocyte apoptosis/survival

Recent evidence shows that cardiomyocyte apoptosis appears to play a major role in many alcoholic cardiomyopathies leading to heart failure (Fernandez-Sola et al., 2006). Specifically, alcohol seems to decrease the activation of a very important survival pathway, Akt (Aroor et al., 2002), as well as that of the anti-apoptotic pathway related to the Mitogen Activated Protein kinases (MAPK), in cardiac myocytes (Table 2; Zhang et al., 2003; Aroor and Shukla, 2004; Li et al., 2006). Such effects have been shown to be agonistic and time dependent, showing an early activation with acute exposure, shifting into deactivation after chronic exposure (Table 2; Li et al., 2006). This is consistent with the J-shaped inverse association between alcohol and cardiovascular disease morbidity and mortality (Panagiotakos et al., 2001; Sempos et al., 2003; Aistrup et al., 2006). Furthermore, acute exposure to high-doses of ethanol treatment induces cardiomycyte apoptosis in a concentration-dependent manner (Altura et al., 1996). High alcohol consumption is associated with myocyte metabolic changes such as decrease in respiratory enzyme and lactate dehydrogenase activity, a decrease in beta oxidation of fatty acids, increase in alcohol dehydrogenase activity which may lead to acetaldehyde accumulation and impaired protein synthesis eventually leading to myocardial injury (Guo et al., 2012).

Sparagna et al. has shown that low-dose alcohol attenuates apoptosis in neonatal rat cardiocytes through Akt and AMP-activated kinase (Sparagna et al., 2004). Converging data are implicating the weakening of the insulin-like growth factor-1 (IGF-1)/PI3 Kinase pathway as an important mechanism of alcohol induced injury (Table 2; Chen et al., 2000). Work from others and our laboratories have shown that the survival benefits of IGF-1 are mainly mediated through PI-3K and to a lesser extent through the MAPK (Teos et al., 2008; Umoh et al., 2014). We have previously shown that PI-3K-dependent IGF-1 signaling activates downstream mTOR pathway in cardiac hypertrophy (Bamji and Haddad, 2015). In addition, IGF-1 receptors and IGF-1 mRNA levels were not affected by chronic alcohol treatment of rats (Pecherskaya et al., 2002). However, there was a higher basal level of IGF-1 receptor activation in alcoholic cardiac protein preparations. Works from other laboratories indicates a negative regulation of I_K_, by IGF-1 mainly through PI-3K/Akt activation (Teos et al., 2008). This may imply that the alcohol-induced depression in I_K_ reported earlier maybe mediated by IGF-1-dependent signaling pathway.

## Low-dose alcohol beneficial cardiovascular effects

Even though long-term alcohol abuse has been associated with defect in cardiac contractility and eventual development of dilated cardiomyopathy and low-output heart failure (Table 2; Preedy et al., 2003), interestingly however, low alcohol consumption has been associated with reducing risk of heart disease and stroke (Berger et al., 1999; Djousse et al., 2004b). Although several hypotheses have been postulated for alcoholic cardiomyopathy and for the low-dose beneficial cardiovascular effects, the precise mechanisms and mediators remain largely undefined. There is a remarkable lack of data in the literature regarding the electrophysiological alterations associated with different levels and length of alcohol exposure in cardiomycytes.

Most of the experimental evidence indirectly relates chronic alcohol-dependent changes in intracellular Ca^2+^ (${\text{Ca}}_{\text{i}}^{2+}$) to contraction dysfunction without assessing the density and/or activity of neither the Ca^2+^ channels nor their inactivation parameters. Interestingly, no data is available on the electrophysiological effects of low alcohol exposure or on the exposure frequency. Clinically relevant concentrations of ethanol induced elevation of ${\text{Ca}}_{\text{i}}^{2+}$ that was dependent on voltage-gated entry into the myocytes through I_Ca,L_ (Brown et al., 1996; Solem et al., 2000) and reduction in the amplitude of K^+^ currents of rats (Nakamura et al., 1999; Dopico, 2003; Liu et al., 2003). It was shown that the density of dihydropyridine binding sites were greater in cardiomycytes isolated from ethanol-consuming rats as compared to control groups (Brown et al., 1996). On the other hand, acute exposure to ethanol has been shown to depress cell shortening and Ca^2+^i in a concentration dependent manner (Ren, 2007). Interestingly, acute exposure to low dose of ethanol reduced the contraction amplitude of the adult rat cardiomycytes; while such effect was associated with lower ${\text{Ca}}_{\text{i}}^{2+}$ when exposed to high doses. Moreover, the beneficial effect of low-dose alcohol was also attributed in part to the fact that ethanol is metabolized differently at low and high concentrations. It was suggested that low ethanol intake has the ability to increase antioxidant capacity, however, at high concentrations ethanol is metabolized to acetaldehyde without producing reduced nicotinamide adenine dinucleotide (NADH). Instead, this pathway utilizes reduced nicotinamide adenine dinucleotide phosphate (NADPH), another reducing equivalent, thus producing an oxidative environment (Lieber, 1990). Interestingly, several studies reported beneficial effects with supplementation of antioxidants in people with essential hypertension and atherosclerotic endpoints (Rimm et al., 1993; Stephens et al., 1996).

## Relationship between inflammatory markers and alcoholic heart disease

Although once thought of as part of inflammatory cells only, cytokines and chemokines are now recognized to play pivotal role in cardiac homeostasis and repair (Tarzami et al., 2002, 2003, 2005; Jougasaki, 2010; LaRocca et al., 2010). Ever since, studies postulated the important role of cytokines in cardiovascular diseases (Ferrari, 1999). It has been found that cytokines are continuously produced, however, they are upregulated in oxidative stress status of the heart. Furthermore, the level of circulating cytokines was inversely associated with left ventricular function (Aukrust et al., 1998). For example, Interleukin-8 was one of the first chemokines to be related to myocardial injury (Oz et al., 1995; Husebye et al., 2014). Similarly, Chemokines (c-c motif) Ligand2 (CCL2) and CCL5 are tremendously involved in heart failure and the death of injured myocardial cells, respectively (Braunersreuther et al., 2010; Abe et al., 2014). Nevertheless, the exact role different cytokines in cardiac pathology remains a largely disputable issue among scientists.

The relationship between inflammation and inflammatory markers with alcoholic heart disease has gained much attention lately. In an *in vivo* study on pregnant Wistar Rats, the authors extrapolated that the inflammation and oxidative stress are the mechanisms of the destructive effect of ethanol on their hearts (Shirpoor et al., 2015). Furthermore, association studies have undoubtedly affirmed the presence of fluctuating levels of inflammatory markers in alcoholic heart disease. For example, IL-6,−8,−12, TNF-α and its receptors-TNF-R were high in patients with alcoholic cardiomyopathy; the higher the severity of the cardiomyopathy the higher the level of these markers (Panchenko et al., 2015). Moiseev and colleagues found the same results in patients with congestive heart failure who had a previous history of alcohol-induced cardiac damage compared to patients who have ischemic cardiomyopathy (Moiseev et al., 2013).

Among these inflammatory markers, TNF-α was particularly interesting for its role in cardiovascular diseases (Ferrari, 1999). In addition to its proinflammatory effect, TNFα has a negative inotropic action on cardiomyocytes (Torre-Amione et al., 1996). In addition to mast cells, which are a notable source of TNFα, the heart is a TNF-producing organ (Gordon et al., 1990; Meldrum et al., 1998). Unlike during physiological conditions, where TNFα levels are low, during heart failure both myocardial macrophages and cardiomyocytes produce a plethora of TNFα; during congestive heart failure, for example, TNF-α were found to be elevated in advanced stages (Meldrum et al., 1998; Torre-Amione et al., 1999; Feldman et al., 2000). Such an effect of TNF-α was consistent with the previous findings on the role of TNF-α in modulating peripheral resistance and the contractility of cardiomyocytes; two core factors that control hemodynamics of the heart function and the circulation (Ferrari, 1999).

The locally produced autocrine TNF-α was found to have a noticeable role in alcohol-related heart failure (Meldrum et al., 1998). As a matter of fact, it was postulated that high level of TNF-α could cause severe cardiac pathologies and participate in changes such as remodeling, fibrosis, and apoptosis (Bryant et al., 1998). Nonetheless, it remains controversial whether TNF-α causes favorable or detrimental effects on the cardiovascular system (Cacciapaglia et al., 2014).

## Therapeutic approaches to improve alcohol-inflicted organ damages

Alcoholism is a multifactorial disorder that requires a multidisciplinary approach to treat depending on the organs affected. Heavy drinkers suffer from many organ damages; among the most effected organs are liver and kidney. As we mentioned before, heart and brain can be affected either directly or indirectly by alcohol and or its breakdown metabolites. So far, conventional treatment strategies have used a combination of a reduction of ethanol-dependent inflicted damage by control drinking and increasing local and systemic protective mechanisms of the body by using antioxidant supplementation (Mailloux, 2016). In the cases of alcohol induced cardiovascular damage the use of anti-inflammatory (Doe et al., 2007; Panchenko et al., 2015), anti-fibrotic and anti-apoptotic (Yang et al., 2013) agents was also suggested.

Other non-conventional suggested approaches included the use of microRNAs since they are shown to play an important role in multi-organ alcohol-induced damages including brain and heart (Natarajan et al., 2015). Altered expression of circulation microRNAs in response to alcoholism has been reported, it was suggested that circulating microRNAs could serve as biomarker and prognostic marker for alcoholism and the degree of damages (Chen Y. J. et al., 2013; Chen Y. P. et al., 2013; Roderburg and Luedde, 2014; Jing et al., 2015). Altered expressions of microRNAs were noted in patients with alcoholic cardiomyopathy and/or brain injury (Jing et al., 2015). The importance of alcohol induced-microRNAs in regulating proinflammatory cytokine e.g., TNF-α and subsequent activation of immune-mediated reaction were also reported (Willeit et al., 2013). miR-155 expression is induced by alcohol and causes activation of NFkB and subsequent increase in TNF-α production in kupffer cells and macrophages, treatment with anti-sense-miR-155 prevented the TNF-α production. Interestingly, ethanol-induced increase in TNF-α expression in the brain was due to an increase in the expression of miR-155. Mice deficient in miR-155 did not show any increase in proinflammatory cytokine levels (e.g., TNF-α) in the brain following ethanol exposure. This suggests an association between certain microRNAs and proinflammatory cytokine (e.g., TNF-α), in ethanol consumption induced neuroinflammation and subsequent brain injury (Lippai et al., 2013).

## Role of TNF-α in alcohol-induced heart damage

Alcohol consumption effects cardiovascular system and predisposes to the development of cardiac abnormalities such as cardiac remodeling, cardiac arrhythmia, cardiomyopathy myocardia infarction, and even SCD (Table 3; Djousse and Gaziano, 2008b; Lai et al., 2011; Xiao et al., 2014; Guzzo-Merello et al., 2015). Consequently, many of these cardiac abnormalities will predispose the patients to development of heart failure thus treatment of alcohol induced heart damages is in accordance with the current clinical treatment of their specific heart condition. Since TNF-α was found to have a noticeable role in alcohol-related heart failure (Meldrum et al., 1998), we are interested in the role of cytokines in particular TNF-α in development and progression of heart failure.

Heavy drinking has been shown to increase the risk of heart failure, thus in addition to the conventional approach to control alcoholism, it is critical to focus on preventive measures that could reduce the risk to heart failure in these patients. It was reported that normal heart does not express TNF but failing heart procures robust amount of TNF-α, Hence, more studies are dedicated to understanding the role of TNF-α in heart failure patients. It is known that TNF-α is elevated in chronic heart failure patient in accordance with their functional class (Heberto Herrera Garza et al., 2002). Such an increase was found to have a linear correlation with the prognosis of chronic heart failure patients (Heberto Herrera Garza et al., 2002). Notably, a concordant reduction in TNF-α receptors on the cardiac myocytes was noticed in heart failure patients too, which was attributed to the high circulating levels of TNF-α. Studies on animal models have shown the harmful effect of the negative inotropic effect of TNF-α on heart failure patients, in whom no transplant was performed (Heberto Herrera Garza et al., 2002). These findings are consistent with changes in TNF-α after improvement of pressure and volume overload, which results from hypertrophic obstructive cardiomyopathy (HOCM) and terminal dilated cardiomyopathy, using ethanol ablation and ventricular assistance, respectively (Heberto Herrera Garza et al., 2002).

Attempts to integrate TNF-α antagonist into the realms of therapeutic medicine has been suggested, with possible effectiveness for symptomatic heart failure patients (Heberto Herrera Garza et al., 2002). Tumor Necrosis Factor Receptor 1 (TNFR1) and TNFR2 are two cell surface receptors for TNF-α, through which it achieves its various outcomes. Yet, variation in expression of these receptors and its regulation during cardiac disease status is not known.

Interestingly, in brain injury, TNF-α has been postulated to potentiate glutamate-mediated cytotoxicity in astrocyte leading to neuronal degeneration (Pickering et al., 2005). Han et al. (2001), have contributed some of the TNF-α cytotoxicity to TNF- mediated stromal-derived factor-1 (SDF-1) activation of NF-κB, during which it increases the production of other cytokines and chemokines and triggers cell death in primary astrocytes (Table 1; Han et al., 2001). Several studies have also linked the expression of TNF-α or SDF-1 to many cardiac pathological conditions, however, a link between TNF-α, SDF-1 and their association with severity of alcohol mediated cardiac disease has not been explored.

SDF-1, otherwise known as CXCL12, plays a major role in regulating stem cell recruitments, inflammation and inflammation mediated injury (Doring et al., 2014; Wang et al., 2014). These roles are made possible mainly through the interactions of SDF-1 and its receptors CXCR4 and CXCR7, with CXCR4 being suggested as the primary receptor due to the fact that selective CXCR4 receptor antagonists block many effects of SDF-1 (Wei et al., 2014). The production of SDF-1 is stimulated by various pro inflammatory stimuli such as TNF-α (Feng et al., 2014). Moreover, TNF-α has been shown to up-regulate SDF-1 expression in cultured astrocytes and endothelial cells (Wei et al., 2014). The expression of SDF-1 has been shown to affect disease process in many cardiovascular diseases.

In the heart, various cells including cardiomyocytes, stromal cells, endothelial cells, fibroblasts, and dendritic cells express SDF-1 (Wei et al., 2014). The wide distribution of SDF-1 in the heart witnesses to its important physiological roles and its roles in disease states. SDF-1 levels are shown to increase in many cardiac pathological conditions including myocardial ischemia, myocardial infarction, myocardial inflammation, atrial fibrillation, and the development of heart failure (Bromage et al., 2014; Wei et al., 2014; Li et al., 2016). Increased expression of platelet SDF-1 was shown to have negative clinical outcomes (Rath et al., 2017). Since, elevated pro inflammatory cytokines such as TNF-α is linked to causing contractile dysfunction, apoptosis and remodeling in the heart (Chen et al., 2010). It could be indicated that elevated levels of both SDF-1 and TNF-α could have a synergistic effect in causing damage in cardiovascular disease.

SDF-1 alpha has also been shown to play roles in other disease conditions that affect other systems in the body commonly affected by heavy alcohol exposure. SDF-1 has been shown to increase in toxic liver damage, neonatal sepsis, autoimmune, inflammatory diseases, *Helicobacter Pylori* induced peptic ulcer, rotator cuff disease, skin inflammation squamous cell carcinoma of the lung, renal ischemia-reperfusion injury, glioblastoma tumor, and AIDS-associated neurologic disorders (Han et al., 2001; Rostasy et al., 2003; Kim et al., 2006; Bromage et al., 2014; Wan et al., 2014; Zgraggen et al., 2014; Seemann and Lupp, 2015; Bagheri et al., 2016; Sterlacci et al., 2016). The binding of SDF-1 to its receptor induces neuronal apoptosis *in vitro*, which indicates the pathological roles of SDF-1 in increasing the severity of neurological impairment due to increased astrocyte cell death (Table 1; Han et al., 2001). Moreover, it has been shown that through astrocytes, SDF-1 inductions of TNF-α provides a course of soluble cytotoxic factors that could induce neuronal cell death and contribute to the pathogenesis of HIV associated dementia (Han et al., 2001). Together, SDF-1 and TNF-α could promote and accelerate local inflammation and damage via their paracrine and autocrine mechanisms (Table 1).

Activation of TNF-α by SDF-1 has also been postulated to promote the reactivation of latent HIV in macrophages and microglial cells (Table 1; Han et al., 2001). Other papers have suggested that TNF-α induction of SDF-1 limits immune cells from entering the CNS (Table 1; Blazevski et al., 2015). However, the protective role for SDF-1 in injury, in particular alcohol-induced injuries are also suggested; when the alcohol induced alteration in circulating chemokines were explored in animal models, they noticed that rats exposed to repeated ethanol had lower SDF-1 and higher Eotaxin-1 (also known as CCL11) concentration in their plasma (Garcia-Marchena et al., 2016). Upregulation of SDF-1 is also associated with reduced size of alcoholic fatty liver transplants in which the action of SDF-1 was contributed to its role in mobilizing of host stem cells to the site of injury and aid in repair and successful transplantation (Hisada et al., 2012). The concerns still remains on whether increases in chemokines and/or cytokines leads to immune activation in remote organs such as the brain and the heart, whether they contribute to subsequent alcohol inflicted injuries, and whether they may have other important functions.

## Conclusion and perspectives

Collectively, these data suggest that the alcohol induced alteration in circulating chemokines is a major contributor in alcoholic mediated organ damages via both direct and indirect mechanisms (Figure 1). The presented data suggests a link between the heavy drinking and an increase in the expression of SDF-1 and TNF-α, however, whether they are involved in alcoholic mediated damage in CNS or heart is not clear and needs further studied. This is important because recently overexpression of SDF-1 via gene therapy was used as a strategy for improving heart failure symptoms in patients with ischemic cardiomyopathy (Penn et al., 2013). In this study, researchers tried to use the body's stem cell-based repair mechanism by drawing the patients' own stem cells to the site of injury using the effect of SDF-1. Even though the results seemed promising, there were some concerns, including the very small group of participants and the lack of placebo group. However, in a follow up study, the final 12-month results from the Phase 2 STOP-HF clinical trial, double-blind, randomized, placebo-controlled trial by Juventas Therapeutics demonstrated that in a population with advanced chronic heart failure who are symptomatic and present with poor cardiac function, a single administration of 30 mg of JVS-100, non-viral DNA plasmid gene therapy of SDF-1, has the potential to improve cardiac function, but failed to demonstrate its primary endpoint of improved composite score at 4 months after treatment (Chung et al., 2015). Stem-cell therapy seems to be a promising approach against alcohol-induced cardiac or non-cardiac systemic damages, however there are a number of hurdles in the path of stem cell research that has prevented the routine application of the technology in regenerative medicine.

In conclusion, alcohol's action are complex, chronic alcohol consumption can increase the risk of heart disease, brain damage, dementia, neuropathy, metabolic disturbances, nutritional deficiencies, certain cancers, liver, and other faces of morbidity and mortality. In this review we have highlighted the contribution of inflammatory process in alcohol-mediated tissue damage and organ dysfunction. What is apparent from literature review is that the full understanding of the related cytokine signaling pathways in alcoholic injuries will be essential for further understanding of their potential contribution and their complex biological effects on alcohol-induced organ damage. Investigation of agents that interfere with inflammatory cytokine production is needed in order to enhance our understanding on their potential contributions into pathogenesis of diseases that are associated with excessive alcohol consumption.

## Author contributions

AO and AP: performed the literature search and designed the tables/figures and edited the manuscript; ST: wrote the manuscript; JL and GH: performed the literature search and discussed the content of the manuscript.

### Conflict of interest statement

The authors declare that the research was conducted in the absence of any commercial or financial relationships that could be construed as a potential conflict of interest.
